# Invisible Facial Flushing in Two Cases of Dengue Infection and Influenza Detected by PC Program and Smartphone App: Decorrelation Stretching and K-Means Clustering

**DOI:** 10.1155/2020/8790130

**Published:** 2020-02-13

**Authors:** Manote Arpornsuwan, Matinun Arpornsuwan

**Affiliations:** ^1^Buriram Hospital, Buriram 31000, Thailand; ^2^Emergency Medicine, Mission Hospital, Bangkok 10300, Thailand

## Abstract

We report the two cases of dengue infection and influenza with invisible facial flushing. The invisible facial flushing can be detected and visible by the Manote and Matinun (M&M) technique using PC program and smartphone app (decorrelation stretching and K-Means clustering). The unique patterns of facial flushing in the patients with high fever provide a clue to the diagnosis of dengue infection and influenza. This new innovative method could detect dengue infection and influenza earlier in the patients with high fever.

## 1. Introduction

Facial flushing, a sensitive and specific predictor of dengue infection [1], was found in approximately half of the dengue-infected patients [2]. Facial flushing was also found in influenza as a physical finding [3]. We developed a new innovative method called Manote and Matinun (M&M) technique applying both the decorrelation stretching and K-Means clustering algorithm to detect invisible facial flushing patterns in dengue infection and influenza.

The decorrelation stretch is a process that is used to enhance (stretch) the color differences found in a color image [4, 5]. It has been used in remote sensing to enhance multispectral images [6, 7].

The National Aeronautics and Space Administration (NASA) has developed a decorrelation stretching method and successfully applied it to enhance the color information of the images and to show up very faint color changes that are almost invisible to the eye. NASA has used it to enhance Mars Rover images [8]. Harman, the American mathematician modified and implemented this technique in the DStretch plugin to ImageJ. The plugin has options intended to be useful in rock art research. It can make pictographs visible that are nearly invisible to the naked eye [9]. Uji et al. from the department of ophthalmology and visual sciences, Kyoto university graduate school of medicine, Kyoto, Japan, used the decorrelation stretching method to successfully enhance color fundus photographs and reveal hidden information in a color fundus photograph [10].

Image segmentation is the division of an image into a set of no overlapping regions whose union is the entire image. The purpose of segmentation is to break down the images into parts that are useful with respect to a particular application [11]. Image segmentation is the classification of an image into different groups. K-Means clustering-based image segmentation algorithm is used to segment the interest area from the background [12].

Four steps of the Manote and Matinun (M&M) technique were used with PC program and smartphone app (decorrelation stretching and K-Means clustering).

  Step 1 (original photograph): take a photo of the patient's face with the flash on by the smartphone camera app
  Step 2 (1^st^ decorrelation stretching picture): decorrelation stretching the face photos using the YUV color space by the PC program ImageJ with its plug-in software DStretch [13] that was originally designed for analyses of rock art
  Step 3 (K-means clustering picture): after decorrelation stretching, perform the K-Means clustering algorithm (*n* = 3) with the EigenCAM android app (3 clusters: (1) Green, (2) Red, and (3) Blue) choosing the red cluster [14]
  Step 4 (2^nd^ decorrelation stretching picture): repeating decorrelation stretching again using the Lab color space (a different color space enhancement from the first one) by the PC program ImageJ with DStretch algorithm plugin

The percentages of facial flushing in dengue infection were observed varying in different studies [2, 15] and based on the naked eye, which were dependent on multiple factors such as races, skin color, skin pigmentation, anemia status, and observer bias. Hemoglobin is what makes blood red, which in turn makes the face flushed. So, for the patients with an iron deficiency or thalassemia, a drop in hemoglobin levels can make it difficult to see facial flushing with the naked eye.

Four steps of the Manote and Matinun (M&M) technique could help the naked eye see the patterns of facial flushing that has not been visible before in case of dengue infection and influenza.

We expected that this new innovative method could detect dengue infection and influenza earlier by distinguishing unique patterns of facial flushing in the patients with high fever and could be a new tool detecting the presence of dengue fever early on, helping prevent people from suffering potential life-threatening complications.

In this case report, we demonstrate the clinical relevance of direct application of both the decorrelation stretching and K-Means clustering method to the original photograph of the patient's face with the smartphone camera app. This is the first case report of invisible facial flushing in dengue infection and influenza detected by PC program and smartphone app. (decorrelation stretching and K-Means clustering).

## 2. Case Reports

Patient 1 was a 13-year-old girl with no known chronic illness presented to the clinic Dr. Manote in Buriram, Thailand, with a history of high fever and fainting beginning two days prior to her presentation. The fever lasted two days and settled with the use of paracetamol. She then started to experience coryza, sore throat, and cough which lasted for two days of his illness.

At the time of presentation, there was no history of muscle pain, neck stiffness, or photophobia. There were no other urinary symptoms and no history of nausea or vomiting. Of note, she gave no history of exposure to influenza recently.

On physical examination, she had normal blood pressure with a temperature of 38.5°C, heart rate 123/minute, respiratory rate 34/minute, and oxygen saturation (SpO_2_) 96-97%. Her cardiovascular, respiratory, abdominal, musculoskeletal, and central nervous systems were all normal. Examination of the skin did not reveal any petechiae, purpurae, ecchymoses, or rash. There was no facial flushing seen by the naked eye. A tourniquet test was performed and was negative.

**Figure 1:**
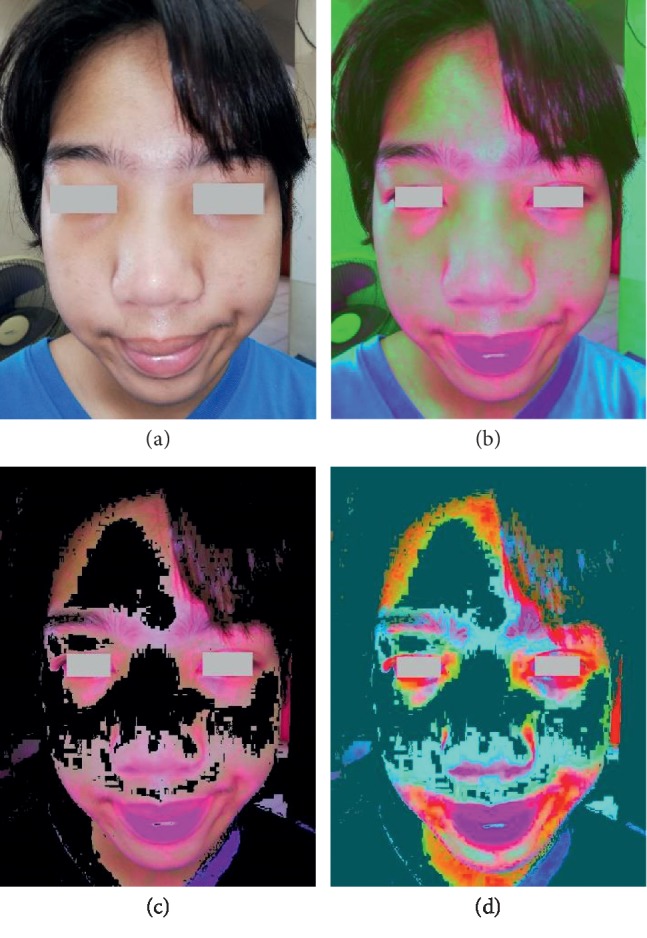
Four steps of the Manote and Matinun (M&M) technique for the 1^st^ patient. (a) The original photograph by the smartphone camera app revealed invisible facial flushing seen by the naked eye. (b) The 1^st^ decorrelation stretching picture using the YUV color space. (c) The K-means clustering picture choosing the red cluster. (d) The 2^nd^ decorrelation stretching picture using the Lab color space.

Four steps of the Manote and Matinun (M&M) technique to this patient revealed localized areas of facial flushing, especially on nose, around the eyes, and forehead which were suggestive of influenza (Figure 1).

Investigations from the private laboratory revealed a negative test for influenza A and a positive test for influenza B by rapid influenza test.

She was treated with oseltamivir 75 mg PO bid for 5 days and gradually improved to full recovery about 5–7 days after treatment.

Patient 2 was a 5-year-old boy who presented with 18 hours of continuous high fever, intermittent abdominal pain, and retro-orbital headache. This patient is the younger brother of the previous case who was diagnosed with influenza B. Two days after his sister's visit, he developed a high fever, accompanying symptoms such as coryza, sore throat, and cough. There were no other urinary symptoms and no history of nausea or vomiting. There had been one episode of epistaxis in ten days before this illness. He had been apparently a healthy child without any significant illnesses in the past.

On physical examination, he had a temperature of 39°C, heart rate 130/minute, and oxygen saturation (SpO_2_) 97-98%. Examination of the skin did not reveal any petechiae, purpurae, ecchymoses, or rash. There was no facial flushing seen by the naked eye. A tourniquet test was performed and was positive.

**Figure 2:**
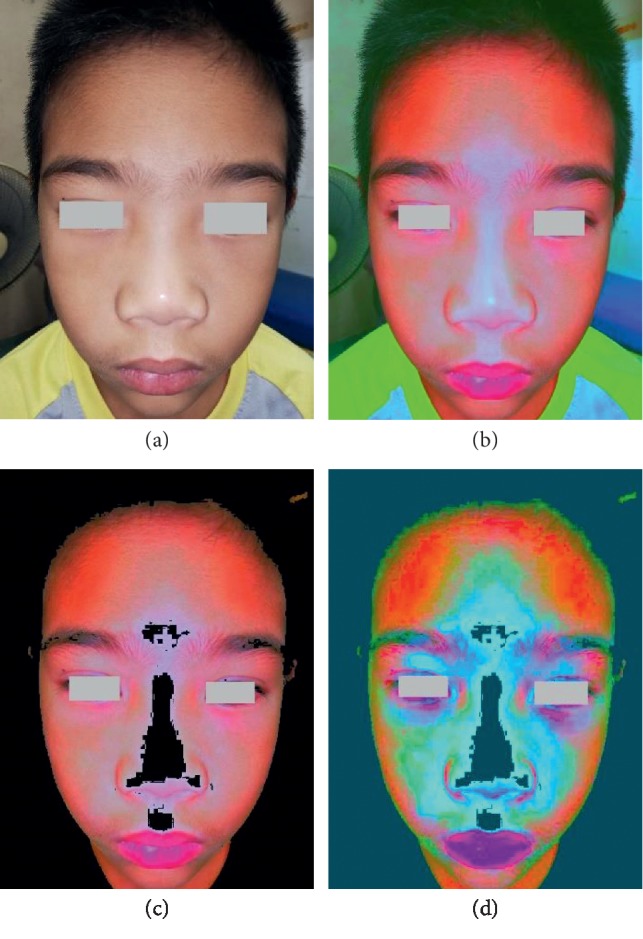
Four steps of the Manote and Matinun (M&M) technique for the 2^nd^ patient. (a) The original photograph by the smartphone camera app showed invisible facial flushing seen by the naked eye. (b) The 1^st^ decorrelation stretching picture using the YUV color space. (c) The K-means clustering picture choosing the red cluster. (d) The 2^nd^ decorrelation stretching picture using the Lab color space.

Four steps of the Manote and Matinun (M&M) technique to this patient revealed generalized areas of facial flushing, including on nose, around the eyes, cheeks, forehead, and perioral area which were suggestive of dengue infection (Figure 2).

Investigations from the private laboratory revealed the presence of IgM antibodies and absence of both IgG antibodies to dengue virus and dengue NS1 antigen. A rapid influenza test revealed a negative test for Influenza A and a positive test for Influenza B.

Recent dengue infection with influenza coinfection was diagnosed by a clue of generalized areas of detecting facial flushing accompanying with symptoms and history of influenza B exposure.

The patient was referred to the Buriram hospital and was admitted two days later. In-hospital management included oseltamivir treatment for influenza, adequate hydration with intravenous and oral fluids, and close monitoring of electrolyte and platelet count. The initial laboratory investigations revealed CBC: Hct 33% WBC 3,200/mm^3^, Platelet count 217,000/mm^3^, Neutrophile 21, Lymphocyte 73, Monocyte 5, Eosinophil 1, Electrolyte: Na 139 mEq/L, K 2.86 mEq/L, Cl 109 mEq/L, HCO_3_ 18 mEq/L.

The hypokalemia was corrected, and the potassium (K) level was increased to the normal limit (K 3.89 mEq/L) on the next day. The lowest platelet count was reported 105,000/mm^3^ on day 6 of the illness and increased to 135,000/mm^3^ on the next day. His vital parameters and serial hematocrit readings remained stable without signs of fluid leakage. The patient's hospital course was uncomplicated, and he was discharged from the hospital with complete recovery.

## 3. Discussion

Flushing is a phenomenon of transient vasodilatation, which is part of a coordinate physiologic thermoregulatory response to hyperthermia, resulting in increased cutaneous blood flow. Various benign and malignant entities may cause flushing. The most common reasons for flushing are fever, hyperthermia, menopause, emotional blushing, and rosacea [16]. But, the most likely causes of high fever with facial flushing are dengue infection [1, 2], influenza [3], and scarlet fever, which usually has a pale area around the mouth called circumoral pallor [17].

In dengue-endemic but resource-limited countries, it may not be practical to screen all patients presenting with acute undifferentiated fever with the dengue NS1 antigen or dengue nucleic acid detection tests, especially if a complete blood count (CBC) performed did not reveal the presence of leukopenia [18].

Dr. Suneth Weerarathna, Senior Registrar in Medicine, Postgraduate Institute of Medicine, Colombo, Sri Lanka, suggested that the early diagnosis of dengue infection with 1–3 days of high fever can be seen in patients with symptoms of high fever and flushed without coryza. This will help to identify dengue infection in the first 1–3 days of fever with sensitivity of 73.3–90.5% and specificity of 87.9–93.3% [19].

Generally, if we look for facial flushing in patients with suspicion of dengue fever or influenza with the naked eye, it will be difficult to see easily, as the skin color on the faces of different patients is obscured for us to be clearly observed.

Decorrelation stretching is an image-processing algorithm which originated in the world of satellite and aerial mapping. Its intended use is to highlight differences in an image that are too subtle for a human to see. Decorrelation stretching is used to enhance color differences in images with high interchannel correlation. Therefore, it allows us to see details that are otherwise not so obvious or invisible to the human eye.

So, if we apply the 1^st^ decorrelation stretching with the face photos of all the patients with dengue infection, including both dengue fever and dengue hemorrhagic fever using the YUV color space by the PC program ImageJ with its plug-in software DStretch, we may detect all cases with invisible facial flushing to visible more than usual.

The YUV color space is “derived” from the RGB space. It comprises the luminance (Y) and two color difference (U, V) components. The luminance can be computed as a weighted sum of red, green, and blue components; the color difference, or chrominance, components are formed by subtracting luminance from blue and from red. The principal advantage of the YUV model in image processing is decoupling of luminance and color information. The importance of this decoupling is that the luminance component of an image can be processed without affecting its color component [20].

Segmentation is a fundamental process in digital image processing which has found extensive applications in areas such as medical image processing, compression, diagnosis arthritis from joint image, automatic text hand writing analysis, and remote sensing. The clustering methods can be used to segment any image into various clusters based on the similarity criteria like color or texture [21].

Utilizing K-means clustering (*n* = 3) to extract the colors from the 1^st^ decorrelation stretching picture choosing the red cluster allows us to see different, unique patterns of the facial flushing between dengue infection and influenza. In cases of influenza, the patterns show localized areas of facial flushing, especially on nose, around the eyes, and forehead in comparison with dengue infection, and they show more generalized areas of facial flushing including on nose, around the eyes, cheeks, forehead, and perioral area.

The final step shows the 2^nd^ decorrelation stretching picture using the Lab color space (different color space enhancement from the first one) after the K-means clustering. The CIELAB color space (also known as CIE L∗a∗b∗ or sometimes abbreviated as simply “Lab” color space) is a color space which expresses color as three values: *L*^*∗*^ for the lightness from black (0) to white (100), *a*^*∗*^ from green (−) to red (+), and *b*^*∗*^ from blue (−) to yellow (+). Lab color is designed to approximate human vision. It aspires to perceptual uniformity, and its *L* component closely matches human perception of lightness [22]. It will enhance the subtle differences in hue again and presented clues for the recognition of the existence and distribution of facial flushing in dengue infection and influenza.

If the sensitivity of this method is more than 90–95%, it can be a good screening tool for the dengue infection in the patients with high fever.

It is difficult to distinguish dengue fever from other febrile illnesses in a dengue-endemic area. Differentiating between dengue fever and influenza based on clinical features alone can be difficult and require laboratory confirmation [23].

The patient 2 was an interesting case because we could diagnose as both dengue with influenza coinfection early in 18 hours after the high fever. The possibility of dengue and influenza coinfection should be considered in locations where these two viruses' epidemic periods coincide to avoid fatal outcomes [24]. Both dengue and influenza are potentially fatal diseases, and a high index of suspicion is necessary among the treating physicians for early diagnosis and best outcomes. Influenza and dengue virus coinfection impairs monocyte recruitment to the lung, increases dengue virus titers, and exacerbates pneumonia [25]. The possible clues for suspicious diagnosis include (a) acute febrile illness, (b) copresentation between respiratory and bleeding symptoms, (c) CBC show lymphocytosis with atypical lymphocytosis and decreased platelet, (d) abnormality of chest X-ray, and (e) history of living in or visiting to a dengue endemic area [26].

In this case, we can recognize and early diagnose dengue with influenza coinfection by clues of generalized areas of detecting facial flushing from the Manote and Matinun (M&M) technique accompanying with symptoms and history of influenza B exposure.

Advantages of the Manote and Matinun (M&M) technique.

1. Noncontact and noninvasive; only take photos with a smartphone
2. Can be very helpful to consider helping make a sound decision for any laboratory tests like a rapid diagnostic test of dengue (NS1Ag, IgG, and IgM) or rapid influenza test.
3. Can be the effective clues for the recognition and early diagnosis of dengue infection and influenza
4. Interpret the findings quickly within 5–10 minutes, and do not wait for half an hour to 1 hour, like waiting for the blood test or rapid influenza results
5. Can be performed repeatedly in 1–3 days after high fever again and without any pain and losing more money like a laboratory tests
6. Can be used in all healthcare facilities or telemedicine following the doctor's advice

In this case report, we have conﬁrmed the signiﬁcant effect of the decorrelation stretching method and its potential clinical relevance. The Manote and Matinun (M&M) technique used with both the decorrelation stretching and K-Means clustering method was successful in enhancing the subtle differences in hue and presented clues for the recognition of the existence and distribution of invisible facial flushing in dengue infection and influenza.

## 4. Conclusion

The Manote and Matinun (M&M) technique used with both the decorrelation stretching and K-means clustering method has the potential to detect dengue infection and influenza earlier by distinguishing unique patterns of facial flushing in the patients with high fever. It is effective, economical, and readily available. Further research and optimization of this method is required for the effective clues for the recognition and early diagnosis of dengue infection and influenza, especially in some health-care facilities with lack of laboratory support.
